# Successful management of sternomanubrial joint septic arthritis with pectoralis muscle flap closure: a case series

**DOI:** 10.1093/jscr/rjaa035

**Published:** 2020-04-29

**Authors:** Barkat Ali, Anil Shetty, Gregory Borah, Christopher Demas, Jess D Schwartz

**Affiliations:** 1 Department of Surgery, Division of Plastic and Reconstructive Surgery, University of New Mexico, Albuquerque, NM; 2 Department of Surgery, Division of Thoracic and Cardiovascular Surgery, University of New Mexico, Albuquerque, NM

**Keywords:** Sternomanubrial joint, SMJ, sternectomy, pectoralis muscle flap closure, PMFC

## Abstract

Primary infection of the sternomanubrial joint (SMJ) is extremely rare. We present four consecutive cases who were all treated with SMJ resection (partial sternectomy), bilateral partial 2nd rib resection and immediate placement of temporary wound vacuum therapy followed by pectoralis major muscle flap closure. Average patient age was 35.5 years with male predominance (75%). All patients had intravenous drug use as underlying risk factor along with concomitant viral infections Hep C (75%) and HIV (25%). MSSA was identified in resection cultures in 75% of the patients. Delayed bilateral PMFC was achieved in all patients (average post-resection day 5). Response to treatment was excellent with no recurrent infections, no complications and zero 30-day mortalities. Our experience represents the largest reported case series in adults and would suggest that aggressive surgical resection followed by PMFC would appear to be the preferred treatment for all patients with SMJ infection.

## BACKGROUND

Primary infection of the sternomanubrial joint (SMJ) is an extremely rare clinical event and is clinically distinct from sternoclavicular joint infections and post-operative sternal wound osteomyelitis [1–3]. Management is controversial and is based on several individual patient case reports [4]. Outcomes with conservative antibiotic therapy or simple incision and drainage alone have been inconsistent; therefore our institution has adopted an aggressive surgical approach to the management of this disease [2]. We report here our outcomes in a series of four patients treated with complete SMJ resection, partial sternectomy, temporary negative pressure wound vacuum therapy and delayed pectoralis muscle flap closure to fill the dead space and cover the defect. We sought to characterize the current presentation of this disease and our experience with this approach in the management of this rare condition.

## CASE PRESENTATIONS

We identified four consecutive patients with the diagnosis of sternomanubrial joint infection from 2005 to 2019. Average age was 35.5 years, (range 29–46) with male predominance of 3:1. All of our patients presented with pain and swelling but not all with leukocytosis. All of our patients had IVDU as the underlying risk factor along with concomitant viral infection hepatitis C (75%), HIV (25%), polysubstance abuse (50%) and sexually transmitted disease (25%). Diagnosis was made with CT scan of chest that demonstrated sternomanubrial joint destruction. All the patients were started on broad spectrum antibiotics empirically followed by resection of the sternomanubrial joints, partial sternectomy and bilateral partial 2nd ribs. Following resection, no mediastinal structures were exposed. The patients were placed in temporary negative pressure wound vacuum therapy with standard pressure settings i.e. 125 mm of Hg until the wounds were ready for closure at which point successful closure was achieved with bilateral pectoralis major muscle flaps, average post-resection day 5. The average number of operations required was 2.25. The antibiotics were tailored on final cultures which were MSSA in three cases (treated with cefazolin) and MRSA in one (treated with daptomycin because of allergy to vancomycin). Response to treatment was excellent with no recurrent infections, no complications and zero 90-day mortalities based on follow-up which averaged 10 months (Table 1).

**Table 1:** Patient characteristics

	Patient 1	Patient 2	Patient 3	Patient 4
Age	46	29	32	35
Gender	M	M	F	M
Presentation	Pain, swelling	Pain, swelling, fever	Pain, swelling, fever	Pain, swelling, fever
Leukocytosis	Yes (11.3)	Yes (11.6)	Yes (14.5)	No (3.7)
Comorbidities	IVDU, Hep B and Hep C	IVDU, Hep C	IVDU, polysubstance abuse, Hep C,	IVDU, polysubstance abuse, HIV, gonorrhea, syphilis
Imaging	CT scan	CT scan	CT scan	CT scan
Surgical debridement	SMJ and 2nd rib	SMJ and 2nd rib	SMJ and 2nd rib	SMJ and 2nd rib
Temporary wound vac	Yes	Yes	Yes	Yes
Wound reconstruction	Bilateral pectoralis major muscle	Bilateral pectoralis major muscle	Bilateral pectoralis major muscle	Bilateral pectoralis major muscle
Placement of antibiotic beads	Yes	Yes	Yes	Yes
Application of incisional wound vac	Yes	Yes	No	No
Days from debridement to closure	7	4	5	4
Number of serial debridements after initial debridement	1	0	0	0
Total number of surgeries	3	2	2	2
Final tissue cultures	MSSA	MSSA	MSSA	MRSA
Antibiotics	Cefazolin	Cefazolin	Cefazolin	Daptomycin
Hospital stay (days)	9	14	13	18
Follow-up (months)	18	4	2	6

## DISCUSSION AND CONCLUSIONS

The sternomanubrial joint lies between the manubrium and the sternal body; it is usually a symphysis, with fibrocartilage covering the bone ends and eventually ossifying later in life [4].

**Figure 1:**
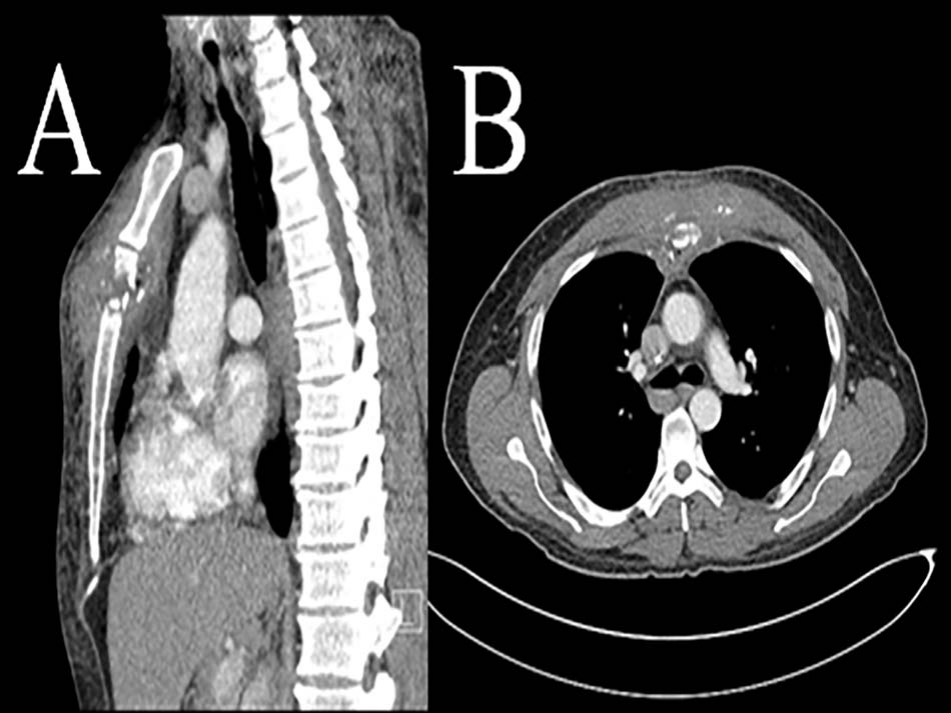
Computed tomography (CT scan) sternomanubrial joint bone erosion and phlegmon. Computed tomography (CT scan): A (sagittal view) and B (axial view).

Primary infection is rare with exact incidence not known. Since first reported in 1976 up until 2019, there have been 14 case reports of sternomanubrial joint infections with a total of 19 patients. Thirteen of these cases are in adults while one paper has described six cases in children [6]. The most common etiology is methicillin-sensitive *Staphylococcus aureus*. There is only one previous paper that has described this disease in IVDU [7]. The underlying risk factors are the same as any other septic arthritis i.e. immunosuppression, steroid use, antimetabolites, concomitant infections distant from the sternomanubrial joint and *Mycobacterium tuberculosis* [4–8]. Pain and swelling over the joint are the most common presenting symptoms. Sometimes, the presentation can be delayed [3]. Fever and leukocytosis are not always present. Diagnosis is based on physical exam and confirmed with imaging using computed tomography (CT scan) or magnetic resonance imaging (MRI). If abscess is found on imaging, joint aspiration can also be performed to help direct preoperative antibiotic treatment (Fig. 1).

A variety of treatment options from IV antibiotics alone to surgical debridement with joint resection and eventual wound closure using muscle flap have been reported. Out of 19 cases reported in the literature, 13 patients have been treated with intravenous antibiotics alone, while four patients have undergone surgical debridement in addition to intravenous antibiotics and wound closure by secondary intention and only two patients have been treated with intravenous antibiotics, resection of the joint and closure with muscle flap [4–8].

Since this is a rare disease, there is no consensus on preferred surgical treatment. Due to the extensive infection and bony involvement in our patients, we performed resection of the joint, any associated ribs and soft tissues that appear to be grossly infected. The resulting defects in our patient series tended to be quite large in size with a deep dead space which was eventually closed with bilateral pectoralis muscle flap. The pectoralis major muscle is well suited for bony coverage of the sternum and mediastinum as is practiced in post cardiac surgery sternal osteomyelitis. It is based on the thoracoacromial pedicle as rotation and/or advancement flap. To add in the mobilization of the muscle, we added release of the pectoralis major muscle tendon on the humeral head.

We report the largest adult series of four patients with primary sternomanubrial joint infections managed with SMJ resection, partial 2nd rib resection and delayed closure and coverage with bilateral pectoralis flaps. All of our patients had 100% procedural success without recurrent osteomyelitis with the treatment plan at an average of 10 months of follow-up. Therefore, we would recommend this approach be undertaken for this rare disease.
